# Searching for the genetic key to a long and healthy life

**DOI:** 10.7554/eLife.57242

**Published:** 2020-04-24

**Authors:** Joris Deelen

**Affiliations:** Max Planck Institute for Biology of AgeingCologneGermany

**Keywords:** lifespan, healthspan, aging, mutation, Human

## Abstract

A study of over 40,000 individuals suggests that carrying a small number of ultra-rare genetic variants is associated with a longer lifespan.

**Related research article** Shindyapina AV, Zenin AA, Tarkhov AE, Santesmasses D, Fedichev PO, Gladyshev VN. 2020. Germline burden of rare damaging variants negatively affects human healthspan and lifespan. *eLife* **9**:e53449. doi: 10.7554/eLife.53449

For centuries scientists have been attempting to understand why some people live longer than others. Individuals who live to an exceptional old age – defined as belonging to the top 10% survivors of their birth cohort – are likely to pass on their longevity to future generations as an inherited genetic trait (van den Berg et al., 2019). However, recent studies suggest that genetics only accounts for a small fraction (~10%) of our lifespan (Kaplanis et al., 2018; Ruby et al., 2018).

One way to unravel the genetic component of longevity is to carry out genome-wide association studies (GWAS) which explore the genome for genetic variants that appear more or less frequently in individuals who live to an exceptional old age compared to individuals who live to an average age. However, the relatively small sample sizes of these studies has made it difficult to identify variants that are associated with longevity (Melzer et al., 2020).

The emergence of the UK Biobank – a cohort that contains a wide range of health and medical information (including genetic information) on about 500,000 individuals – has made it easier to investigate the relationship between genetics and longevity. Although it is not yet possible to study longevity directly with the data in the UK Biobank, several GWAS have used these data to study alternative lifespan-related traits, such as the parental lifespan and healthspan of individuals (defined as the number of years lived in the absence of major chronic diseases). These studies have been reasonably successful in identifying new genetic variants that influence human lifespan, but these variants can only explain ~5% of the heritability of the lifespan-related traits (Timmers et al., 2019; Zenin et al., 2019).

The GWAS have only focused on relatively common genetic variants (which have minor allele frequencies (MAFs) of ≥1%), and it is possible that rare variants might be able to explain what is sometimes called the ‘missing heritability’. Now, in eLife, Vadim Gladyshev (Harvard Medical School) and co-workers – including Anastasia Shindyapina (Harvard) and Aleksandr Zenin (Lomonosov Moscow State University) as joint first authors – report how they analyzed data from the UK Biobank and the UK Brain Bank Network (which stores and provides brain tissue for researchers) to investigate how rare genetic variants affect lifespan and healthspan (Shindyapina et al., 2020).

One type of rare genetic variant, called a protein-truncating variant, can dramatically impact gene expression by disrupting the open reading frame and shortening the genetic sequence coding for a protein. The team calculated how many of these rare protein-truncating variants, also known as PTVs, were present in the genome of each individual, and found ultra-rare PTVs (which have MAFs of <0.01%) to be negatively associated with lifespan and healthspan. This suggests that individuals with a small number of ultra-rare PTVs are more likely to have longer, healthier lives. Stratifying the data by sex showed that the association with healthspan was female-specific, while the association with lifespan was observed in both sexes.

Further analyses revealed that certain types of ultra-rare PTVs (such as stop-gain and frameshift mutations) were more likely to be associated with changes in lifespan. Shindyapina et al. also found that the impact of the variants depended on the damage they caused: for example, if the ultra-rare PTVs resulted in loss-of-function mutations, or if they affected genes that are expressed in many different cell types, the reduction in lifespan was greater. Ultra-rare PTVs were found to be spread across the genome, and only a small group of about 1500 seemingly essential genes did not have these variants. It is likely that damage to any of these 1500 or so genes leads to embryonic lethality or early mortality.

This work is the first to show that rare genetic variants play a role in lifespan-related traits, which is in line with previous studies showing rare PTVs to be linked to a variety of diseases (DeBoever et al., 2018). However, these variants only have a relatively small effect on human lifespan and cannot fully explain how longevity is genetically passed down to future generations. To explain the remaining ‘missing heritability’, future studies should try to focus on gene-by-gene and gene-by-environment interactions.

The UK Biobank is known to have a selection bias towards healthy individuals and the restricted age range of this cohort resulted in most of the individuals studied still being alive at the end of the follow-up period (Fry et al., 2017). Future studies should investigate whether cohorts with a broader age range and more reported deaths (including those of non-European ancestry) can replicate these findings. These studies could also determine whether individuals who live to an exceptional old age (as defined using the criteria outlined in van den Berg et al., 2019) have fewer or complete absence of ultra-rare PTVs.
